# Relationships between patterns of cannabis use, abuse and dependence and recent stimulant use: Evidence from two national surveys in Ireland

**DOI:** 10.1371/journal.pone.0255745

**Published:** 2021-08-09

**Authors:** Seán R. Millar, Deirdre Mongan, Claire O’Dwyer, Bobby P. Smyth, Ivan J. Perry, Brian Galvin

**Affiliations:** 1 Health Research Board, Dublin, Ireland; 2 School of Public Health, University College Cork, Cork, Ireland; 3 Department of Public Health & Primary Care, Trinity College Dublin, Dublin, Ireland; University of the West of Scotland, UNITED KINGDOM

## Abstract

**Background and objectives:**

Epidemiological studies show that the use of cannabis is related to the use of other illicit drugs, including stimulants such as cocaine and ecstasy. However, few studies have examined how patterns of cannabis use relate to the use of stimulants. In this research we determined relationships between patterns of cannabis use and recent stimulant use, drawing on data from two large nationally representative surveys. We also explored how frequency of cannabis use relates to stimulant use and whether subjects with a cannabis use disorder (CUD)–defined as cannabis abuse or dependence–are more likely to be recent users of cocaine or ecstasy.

**Materials and methods:**

We analysed data from Ireland’s 2010/11 and 2014/15 National Drug Prevalence Surveys,which recruited 5,134 and 7,005 individuals respectively, aged 15 years and over, living in private households. We included only those people who reported some past cannabis use. Multivariable logistic regression analysis was used to examine associations between patterns of cannabis use and recent stimulant use.

**Results:**

Among survey participants who had used cannabis in the last month, 17.9% reported recent cocaine use, while almost one-quarter (23.6%) reported recent ecstasy use. There was a significant linear relationship between patterns of cannabis use and recent use of cocaine, ecstasy or any stimulant, with last month cannabis users displaying greater odds (OR = 12.03, 95% CI: 8.15–17.78) of having recent stimulant use compared to last year (OR = 4.48, 95% CI: 2.91–6.91) and former (reference) cannabis users. Greater frequency of cannabis use in the last 30 days was also significantly related to the use of stimulants. In addition, results demonstrated an association between CUD and recent use of cocaine or ecstasy (OR = 2.28, 95% CI: 1.55–3.35).

**Conclusions:**

Findings from this study suggest a relationship between patterns and frequency of cannabis use and recent use of stimulants and an association between CUD and stimulant use. As the use of cannabis with stimulants may increase the risk of negative health consequences, education in community and medical settings about polydrug use and its increased risks may be warranted.

## Introduction

Cannabis is the third most commonly used drug in the world (after alcohol and nicotine) and is the most widely used illicit substance in the Republic of Ireland [1, 2]. Data from a national survey, conducted in 2014/15, showed that 27.9% of Irish people aged 15–64 years had used cannabis at some point in their lives, with 7.7% and 4.4% having used cannabis within the last year or last month respectively [3]. These prevalence figures are considerably higher than those recorded for any other illegal drug and have increased considerably since 2002/03 [2, 4]. In addition, recent data indicate that among Irish people who had used cannabis in the last year, 19.6% met the criteria for cannabis use disorder (CUD) [5]. CUD is now the most common disorder present in new treatment episodes across Europe [6] and there has been a substantial increase in such presentations in Ireland during the past decade [7].

Individuals who use cannabis are more likely to use other illicit substances [8–12], with several epidemiological studies showing that the use of cannabis is significantly related to the use of ‘harder’ illegal drugs, including stimulants such as cocaine and ecstasy [8, 13]. Increasingly, people entering addiction treatment are presenting with polysubstance use [7]; in Ireland over 90% of cocaine users have a history of cannabis use [14]. In addition, treatment data have demonstrated a sharp spike in the number of cocaine users presenting for treatment in Ireland; cocaine accounted for nine percent of new drug treatment cases in 2012, rising to 31% of new treatment cases in 2018 [15]. There have also been noticeable increases in the prevalence of ecstasy use in Ireland; reported last year use of ecstasy decreased between 2006/07 and 2010/11 but increased substantially in 2014/15[2]. Importantly, the simultaneous use of cannabis and stimulant drugs increases the possibility of both adverse mental health and medical effects [16, 17].

Higher rates of polysubstance use among cannabis users may be due to the accessibility and availability of drugs [18], personality traits [19, 20] and the role of cannabis in managing adverse or unpleasant mood states induced by the use of stimulants [11, 21–23]. Nevertheless, despite strong evidence showing an association between cannabis and polydrug use, many authors contend that this relationship may be attributed to other sociodemographic and environmental determinants rather than causal effects of gateway substances [24, 25]. In addition, few studies have examined how patterns of cannabis use relate to the use of stimulants.

Further research on relationships between cannabis and stimulant use is needed owing to the liberalisation of cannabis policies in many world settings. Knowledge of how patterns of cannabis use relate to the use of other substances is important to guide future regulation systems, to inform both clinical and public health practice and for assessing drug policy. This is particularly relevant in Ireland at the present time given the rise in treatment cases presenting for CUD and cocaine use, as well as increases in the use of ecstasy observed among the general population. Therefore, the aim of this study was to determine associations between patterns of cannabis use and recent stimulant use, drawing on data from two large nationally representative studies, which used the same field survey procedures and data collection methods. We also explored the association between frequency of cannabis use and stimulant use and whether individuals with a CUD are more likely to be recent users of cocaine or ecstasy. CUD is an overarching term that combines cannabis abuse and cannabis dependence.

## Materials and methods

### Study population and setting

We analysed data from Ireland’s 2010/11 and 2014/15 Drug Prevalence Surveys. These national, cross-sectional studies recruited stratified clustered samples of individuals aged 15 years and over living in private households in Ireland. The numbers interviewed in 2010/11 and 2014/15 were 5,134 and 7,005 respectively. For both surveys, the sampling frame used was the An Post/Ordnance Survey Ireland GeoDirectory database, which is a list of all addresses in the Republic of Ireland and distinguishes between residential and commercial establishments. A three-stage process was used to construct the sample for each survey. The first stage involved stratifying the population into 10 former health board regions in Ireland. In the second stage of stratification, electoral divisions were selected as the primary sampling units across the 10 former health board regions. Before selection, the primary sampling units were ranked by the following sociodemographic indicators: population density, male unemployment and social class, according to the year of data collection, to ensure that a representative cross-section of areas were included. Finally, in each primary sampling unit, addresses were chosen randomly, and at each address, one person was selected to participate in the survey using the ‘last birthday’ rule. For each survey, the achieved sample was weighted by sex, age and former health board region to maximise its representativeness of the general population. The surveys involved both pre-weights and post-stratification (PS) weights. Pre-weights were used to allow for different selection probabilities of each end respondent, while the PS weights were used to scale up the responses within each demographic cell to represent their respective target populations. A more comprehensive description of the methodology used in the surveys has been detailed elsewhere [26, 27].

The surveys involved face-to-face interviews in the home of each participant and a self-completed questionnaire. The home interviews were conducted by trained interviewers using Computer Assisted Personal Interviewing (CAPI). Fieldwork for the surveys was carried out between October 2010 to May 2011 and August 2014 to August 2015 and both achieved a 60% response rate. All data collection methods were performed in accordance with relevant guidelines and regulations and all data were fully anonymised. No data on non-respondents were collected. The studies were granted ethical approval by the Research Ethics Committee of the Royal College of Physicians in Ireland and signed informed consent was obtained from the parental guardians of minors and from subjects 18+ years of age for data to be used for research purposes. Ireland’s National Drug Prevalence Surveys are GDPR compliant. In this study we included only those participants who reported some past lifetime use of cannabis.

### Outcome variables

#### Recent cocaine, recent ecstasy or any recent stimulant use

Stimulant use was defined via European Monitoring Centre for Drugs and Drug Addiction (EMCDDA) criteria [28]. Respondents who used cocaine or ecstasy at least once within the past year were defined as recent users. Any recent stimulant use was defined as any cocaine and/or ecstasy use in the past year.

### Exposure variables

#### Cannabis use

Patterns of cannabis use were defined via the EMCDDA criteria [28]. Respondents who had used cannabis at some point in their lives were classified into one of three mutually exclusive groups. These groups were former users (used cannabis at least once in their life but not in the past year), recent users (used cannabis at least once within the past year but not in the past month) and current users (used cannabis at least once within the past month). Frequency of cannabis use among current users was determined by asking participants: “During the last 30 days on how many days have you taken cannabis?” Data on frequency of use were recorded for 394 respondents.

#### Cannabis use disorder (CUD)

The Diagnostic and Statistical Manual of Psychiatric Disorders (DSM), better known as the DSM-IV, is published by the American Psychiatric Association and covers all mental health disorders for children and adults. Substance abuse and dependence is defined by the DSM-IV as a maladaptive pattern of substance use leading to clinically significant impairment or distress [29, 30]. In this study cannabis abuse and cannabis dependence were classified according to DSM-IV criteria and were measured via a self-completed questionnaire using the four items that denote cannabis abuse and seven items that denote cannabis dependence from the Composite International Diagnostic Interview (CIDI). Cannabis abuse was established from a positive response in one or more of the four domains on the DSM-IV diagnostic criteria in the 12 months before the interview. Cannabis dependence was determined from a positive response in three or more of the seven domains on the DSM-IV in the 12 months before the interview. CUD was determined as any cannabis abuse or dependence in the 12 months prior to the interview.

The CIDI is a widely accepted and frequently used operationalisation of the DSM-IV. Advised by the EMCDDA, the abbreviated version, the Munich Composite International Diagnostic Interview (M-CIDI), a 19-item instrument reflecting the four cannabis abuse and the seven cannabis dependence criteria, was used for both the 2010/11 and 2014/15 Drug Prevalence Surveys [31].

### Confounding variables

Independent variables likely associated with cannabis and stimulant use were identified through the literature. Demographic variables selected were sex, age and marital status. Additionally, geographic region (Dublin/outside Dublin) was included to control for any possible effects of sociopolitical and cultural factors present within Ireland. Educational attainment, employment and housing tenure were also included as covariates in analyses.

### Statistical analysis

The datasets were codified, affixed with a timestamp variable (year) and merged. Study participants who had never used cannabis in their lives were excluded from analyses. Descriptive characteristics were examined according to recent stimulant use. Logistic regression analyses were used to examine associations between patterns of cannabis use and recent stimulant use. An unadjusted model was used to test associations. A second model was adjusted for sex and age and a third model was adjusted for sex, age, marital status, region, education, employment, housing tenure and year of data collection. Relationships between frequency of cannabis use (defined as a linear variable, ordered tertiles or a categorical variable based on cut-offs from previous research [32]) and recent stimulant use among participants who had used cannabis in the last month, and CUD (defined as either cannabis abuse or dependence) and recent stimulant use among participants who had used cannabis in the last year, were also explored using the methods described above.

Data analysis was conducted using Stata SE Version 15.1 (Stata Corporation, College Station,TX, USA) for Windows. For all analyses, a P-value (two-tailed) of less than .05 was considered to indicate statistical significance.

## Results

### Descriptive characteristics

Characteristics of the 2,979 cannabis users for the full sample, and according to stimulant use, are shown in Table 1. Among subjects who had ever used cannabis, the weighted prevalence of recent cocaine use, recent ecstasy use or any recent stimulant use, was 4.9%, 4.7% and 7.9% respectively; 17.9% of current cannabis users reported recent cocaine use, while almost one-quarter (23.6%) of current users reported recent ecstasy use. In this sample of cannabis users, the prevalence of CUD among recent cocaine and recent ecstasy users, and among participants with any recent stimulant use, was 34.9%, 48.2% and 38.7%.

**Table 1:** Characteristics of cannabis users–full sample and according to recent stimulant use.

Characteristic	Full Sample	No recent stimulant use (any) n = 2743	Recent cocaine use	Recent ecstasy use	Recent stimulant use
	n = 2979		n = 145	n = 141	
					(any) n = 235
**Sex (%)**					
Male	1932 (64.6)	1743 (63.5)	117 (80.7)	103 (73.0)	180 (76.3)
**Age group (%)**					
35+	1354 (45.5)	1319 (48.2)	28 (19.2)	16 (11.3)	35 (14.9)
25–34	1102 (37.1)	1012 (36.9)	68 (46.6)	47 (33.3)	90 (38.3)
15–24	518 (17.4)	408 (14.9)	50 (34.2)	16 (11.3)	110 (46.8)
**Marital status (%)**					
Married/cohabiting	1642 (55.1)	1590 (58.0)	37 (25.5)	23 (16.3)	52 (22.1)
Divorced/separated/widowed	156 (5.2)	151 (5.5)	4 (2.8)	1 (0.7)	4 (1.7)
Single/never married	1178 (39.6)	999 (36.5)	104 (71.7)	117 (83.0)	179 (76.2)
**Region (%)**					
Dublin (compared to outside Dublin)	1133 (38.0)	1028 (37.5)	62 (42.5)	70 (49.6)	105 (44.7)
**Education (%)**					
Completed third level	1677 (56.4)	1544 (56.4)	86 (58.9)	79 (56.0)	133 (56.6)
Completed secondary	998 (33.5)	932 (34.0)	40 (27.4)	39 (27.7)	65 (27.7)
Primary/none	299 (10.1)	262 (9.6)	20 (13.7)	23 (16.3)	37 (15.7)
**Employment (%)**					
Employed	1875 (63.0)	1779 (64.8)	76 (52.4)	47 (33.1)	96 (41.0)
Unemployed	527 (17.7)	454 (16.5)	52 (35.9)	37 (26.1)	73 (31.2)
Student	321 (10.8)	260 (9.5)	14 (9.7)	56 (39.4)	61 (26.1)
Other	255 (8.6)	251 (9.1)	3 (2.1)	2 (1.4)	4 (1.7)
**Housing tenure (%)**					
Owned	1657 (55.6)	1592 (58.0)	46 (31.7)	31 (21.8)	65 (27.7)
Rented	1033 (34.7)	917 (33.4)	72 (49.7)	72 (50.7)	116 (49.4)
Living with family/friends or other	289 (9.7)	235 (8.6)	27 (18.6)	39 (27.5)	54 (23.0)
**Cannabis use (%)**					
Former	2215 (74.4)	2166 (78.9)	42 (29.0)	14 (9.9)	49 (20.9)
Recent	362 (12.1)	308 (11.2)	31 (21.4)	32 (22.7)	54 (23.0)
Current	402 (13.5)	270 (9.8)	72 (49.7)	95 (67.4)	132 (56.2)
**Cannabis use disorder** ^**a**^ **(%)**					
Yes	253 (8.5)	162 (5.9)	51 (34.9)	68 (48.2)	91 (38.7)
**Age at first use (mean)**					
Cannabis	19.16 ± 5.0	19.38 ± 5.1	16.07 ± 2.7	16.40 ± 2.6	16.12 ± 2.7
Cocaine	21.88 ± 5.0	22.27 ± 4.7	20.09 ± 4.4	20.09 ± 3.7	20.23 ± 4.1
Ecstasy	19.90 ± 4.2	20.42 ± 4.4	18.09 ± 3.3	18.51 ± 3.4	18.22 ± 3.2

Displayed frequencies and percentages (in brackets) and means± one standard deviation are weighted. Numbers may not add up in column totals because of missing data.

^a^Any cannabis abuse or dependence.

A higher percentage of recent stimulant users were single/never married, had completed third level education and were living in rented accommodation. Recent cocaine users were more likely to be employed (52.4%), while almost two-fifths (39.4%) of recent ecstasy users were students. Mean age at first use of cannabis was noticeably lower among stimulant users compared to age at first use of cocaine or ecstasy. Mean age at first use of cannabis, cocaine and ecstasy was also lower among subjects who had used stimulants in the last year compared to survey participants who reported no recent cocaine or ecstasy use.

### Relationships between patterns of cannabis use and recent stimulant use

Table 2 displays odds ratios for the associations between recent stimulant use and recent or current cannabis use (compared to former use). A significant linear trend was noted between patterns of cannabis use and recent stimulant use, with current cannabis users displaying greater odds of having recent stimulant use compared to recent and former cannabis users. Associations were noticeably strong. In fully adjusted models, recent cannabis users had three-fold (OR = 3.41, 95% CI: 2.05–5.67), eight-fold (OR = 8.12, 95% CI: 4.09–16.15) and four-fold (OR = 4.48, 95% CI: 2.91–6.91) increased odds of being recent cocaine or recent ecstasy users, or of having any recent stimulant use, respectively. When compared to former cannabis users, participants who were current users were seven times (OR = 7.37, 95% CI: 4.70–11.55), 26 times (OR = 25.94, 95% CI: 13.75–48.91) and 12 times (OR = 12.03, 95% CI: 8.15–17.78) more likely to have used cocaine, ecstasy or any stimulant in the last year.

**Table 2:** Odds ratios for the associations between patterns of cannabis use and recent stimulant use.

Cannabis use patterns		Recent cocaine use		Recent ecstasy use		Recent stimulant use (any)	
		OR (95% CI)	P-value	OR (95% CI)	P-value	OR (95% CI)	P-value
**Model 1**							
	Former	1.00 (Ref.)	< .001^a^	1.00 (Ref.)	< .001^a^	1.00 (Ref.)	< .001^a^
	Recent	4.92 (3.05–7.92)	< .001	15.89 (8.33–30.32)	< .001	7.80 (5.20–11.69)	< .001
	Current	11.30 (7.59–16.82)	< .001	50.78 (28.37–90.89)	< .001	21.72 (15.28–30.88)	< .001
**Model 2**							
	Former	1.00 (Ref.)	< .001^a^	1.00 (Ref.)	< .001^a^	1.00 (Ref.)	< .001^a^
	Recent	3.58 (2.17–5.91)	< .001	9.04 (4.62–17.68)	< .001	4.93 (3.22–7.55)	< .001
	Current	7.89 (5.15–12.06)	< .001	30.92 (16.87–56.65)	< .001	13.98 (9.65–20.27)	< .001
**Model 3**							
	Former	1.00 (Ref.)	< .001^a^	1.00 (Ref.)	< .001^a^	1.00 (Ref.)	< .001^a^
	Recent	3.41 (2.05–5.67)	< .001	8.12 (4.09–16.15)	< .001	4.48 (2.91–6.91)	< .001
	Current	7.37 (4.70–11.55)	< .001	25.94 (13.75–48.91)	< .001	12.03 (8.15–17.78)	< .001

Binary logistic regression. Odds ratios (OR) and 95% confidence intervals (CI) are shown.

Model 1: univariate.

Model 2: adjusted for sex and age.

Model 3: adjusted for sex, age, marital status, region, education, employment, housing tenure and year of data collection.

^a^P-value for linear trend.

### Relationships between frequency of cannabis use and recent stimulant use among current cannabis users

Associations between frequency of cannabis use and recent stimulant use, among current cannabis users, are displayed in Table 3. For the outcome, we compared the fit of a parsimonious model defining frequency of cannabis use as linear predictor variable to models treating it as ordered tertiles or a categorical variable using cut-offs determined in previous research. The latter two fit the model better: LR *x*^2^ = 2.45 (linear) vs. LR *x*^2^ = 17.59 (tertiles) vs. LR *x*^2^ = 17.84 (categorical). After controlling for potential confounders, a 1-category increase in cannabis use frequency was associated with a significant increase in the odds of recent stimulant use (OR = 1.03, 95% CI: 1.00–1.06). In multivariable models, respondents who had used cannabis more frequently within the last month (tertile 2 and tertile 3) had 2.25 (95% CI: 1.22–4.15) and 2.43 (95% CI: 1.33–4.41) increased odds respectively of having any recent stimulant use compared to participants who had used cannabis less frequently (tertile 1, P for trend = .029). Participants who had used cannabis daily 10–19 times per month or 20 or more times per month were 2.5 times more likely to be recent stimulant users.

**Table 3:** Odds ratios for the associations between frequency of cannabis use and recent stimulant use among subjects who had used cannabis within the last 30 days.

Frequency of cannabis use	Recent stimulant use (any) n = 127	No recent stimulant use (any) n = 267	Model 1		Model 2		Model 3	
			OR (95% CI)	P-value	OR (95% CI)	P-value	OR (95% CI)	P-value
Linear	6.0 (4.0–11.7)^a^	4.0 (2.0–10.0)^a^	1.02 (0.99–1.04)	.115	1.02 (1.00–1.05)	.051	1.03 (1.00–1.06)	.047
Tertile 1	29 (22.8)	118 (44.4)	1.00 (Ref.)	.008^b^	1.00 (Ref.)	.007^b^	1.00 (Ref.)	.029^b^
Tertile 2	46 (36.2)	65 (24.4)	2.84 (1.64–4.94)	< .001	2.60 (1.48–4.59)	.001	2.25 (1.22–4.15)	.009
Tertile 3	52 (40.9)	83 (31.2)	2.51 (1.47–4.27)	.001	2.68 (1.55–4.64)	< .001	2.43 (1.33–4.41)	.004
1–3 times per month	29 (22.8)	118 (44.2)	1.00 (Ref.)	.063^b^	1.00 (Ref.)	.044^b^	1.00 (Ref.)	.06^b^
4–9 times per month	46 (36.2)	65 (24.3)	2.84 (1.64–4.94)	< .001	2.69 (1.53–4.74)	.001	2.30 (1.25–4.23)	.007
10–19 times per month	28 (22.0)	41 (15.4)	2.73 (1.46–5.13)	.002	3.06 (1.60–5.84)	.001	2.53 (1.27–5.06)	.009
20+ times per month	24 (18.9)	43 (16.1)	2.29 (1.21–4.35)	.011	2.49 (1.29–4.79)	.006	2.54 (1.23–5.24)	.012

Binary logistic regression. Odds ratios (OR) and 95% confidence intervals (CI) are shown. Displayed frequencies and percentages (in brackets) are weighted.

Model 1: univariate.

Model 2: adjusted for sex and age.

Model 3: adjusted for sex, age, marital status, region, education, employment, housing tenure and year of data collection.

^a^Median and (interquartile range).

^b^P-value for linear trend.

### The relationship between CUD and recent stimulant use

Table 4 shows the association between CUD and recent stimulant use among respondents who had used cannabis within the last year. Among this cohort, almost half (48.9%) of recent stimulant users indicated having a CUD. In a model which adjusted for sex, age, marital status, region, education, employment, housing tenure and year of data collection, study participants with a CUD were found to have a two-fold increased likelihood of having any recent stimulant use (OR = 2.28, 95% CI: 1.55–3.35) compared to last year cannabis users without a CUD.

**Table 4:** Odds ratios for the association between having a cannabis use disorder and recent stimulant use.

Cannabis use disorder^a^	Recent stimulant use (any) n = 186	No recent stimulant use (any) n = 578	Model 1		Model 2		Model 3	
			OR (95% CI)	P-value	OR (95% CI)	P-value	OR (95% CI)	P-value
No	95 (51.1)	416 (72.0)	1.00 (Ref.)		1.00 (Ref.)		1.00 (Ref.)	
Yes	91 (48.9)	162 (28.0)	2.45 (1.75–3.45)	< .001	2.21 (1.56–3.13)	< .001	2.28 (1.55–3.35)	< .001

Binary logistic regression. Odds ratios (OR) and 95% confidence intervals (CI) are shown. Displayed frequencies and percentages (in brackets) are weighted.

Model 1: univariate.

Model 2: adjusted for sex and age.

Model 3: adjusted for sex, age, marital status, region, education, employment, housing tenure and year of data collection.

^a^Any cannabis abuse or dependence.

## Discussion

In this study we used data from Ireland’s 2010/11 and 2014/15 National Drug Prevalence Surveys to determine relationships between patterns of cannabis use and recent stimulant use. We found that 17.9% of current cannabis users reported recent cocaine use, while almost one-quarter (23.6%) reported recent ecstasy use. Our findings demonstrate a significant linear association between patterns of cannabis use and recent use of cocaine, ecstasy or any stimulant, with current cannabis users displaying greater odds of having recent stimulant use compared to recent and former cannabis users. Among subjects who had used cannabis in the last month, greater frequency of use was also significantly related to the use of stimulants. In addition, our results indicate an association between CUD (defined as cannabis abuse or dependence) and recent use of cocaine or ecstasy.

Preliminary findings from the recently completed 2019/20 drug survey in Ireland suggest that while there has been no change in the prevalence of any recent illegal drug use since 2014/15, there have been changes regarding the types of drugs used [5]. Noticeably, the use of stimulant drugs, in particular cocaine and ecstasy, has increased. Similar trends have been observed in Irish treatment data, where there has been a small decrease in treated cannabis use, but a considerable increase in treated cocaine use [33]. Ecstasy was found to be the second most commonly used illegal drug in Ireland in the year prior to the survey (after cannabis); the largest increase in ecstasy use was seen among males aged 25–34 years, with almost 1 in 10 males in this age group having used ecstasy in the last year. A similar proportion of this cohort had used cocaine in the last year. Overall, recent cocaine use was found to have increased significantly among 15–64-year-olds since 2014/15, indicating that cocaine use remains high in Ireland compared to other European countries [2]. Also of note, is that those who reported illegal drug use in 2019/20 were more likely to report the use of two or more illegal substances. In the 2019/20 survey, 25.0% indicated having used at least three illegal drugs in the last year compared to 15.4% in 2014/15.

While initial results from the 2019/20 survey suggests a small decrease in recent use, cannabis remains the most widely used illicit substance in the Republic of Ireland, and its use among males aged 25–34 years has continued to increase [5]. The EMCDDA have noted a positive relationship between the prevalence of cannabis use and stimulants; countries where there is a higher prevalence of cannabis use also typically have higher rates of stimulant use during the same time period [34]. Different theories have been proposed to explain this correlation. The use of stimulants such as cocaine and ecstasy does not exist in isolation; if subjects are using either substance, they are also more likely to be using another drug, suggesting the existence of a common factor underlying the use of both cannabis and stimulants, such as rising disposable incomes among users of illicit drugs [35] or some type of personal characteristic [36].

Though numerous studies have demonstrated associations between cannabis and the use of other illicit substances, fewer studies have examined relationships between patterns of cannabis use and other drugs. Using a cross-sectional sample of 1,075 adults in the north-eastern United States, Tzilos et. al [11] demonstrated that daily cannabis use was associated with higher odds of opiate, stimulant, hallucinogen and inhalant use. In a longitudinal latent class analysis of 5,315 adolescents from the United Kingdom, Taylor et. al [13] found that one-fifth of adolescents followed a pattern of occasional or regular cannabis use, and that these subjects were more likely to progress to harmful substance use behaviours compared to a non-user class. Using participant-level data from three longitudinal studies from Australia and New Zealand, Silins et. al [37] recorded dose-response relationships between the frequency of adolescent cannabis use and adverse young adult outcomes including use of other illicit drugs, high school non-completion, degree non-attainment and suicide attempt.

Several factors may partly explain observed associations between patterns and frequency of cannabis use and the use of other illicit substances including stimulants. These include environmental, behavioural and biological factors. A previous study by our group observed that frequent users of cannabis are more likely to be single and to have a low-level of ownership in living arrangements [4], factors that were also observed among stimulant users in this study. These relationships are likely related to life-course theory [38], as family roles and increased responsibility are generally incompatible with substance use [39]. Also, importantly, we observed that mean age at first use of cannabis was lower than for cocaine and ecstasy among recent stimulant users. Consequently, these findings could be interpreted as a supporting the sequential initiation pattern of cannabis preceding stimulant use [40]. Our previous study also demonstrated that subjects who used cannabis earlier were more likely to be current users of cannabis and to be using it more heavily [40]. It is possible that the availability and accessibility of cocaine and ecstasy increase as subjects use cannabis more frequently, as the illegal status of all three substances likely increases purchasing opportunities for people who use cannabis [11, 18]. Also, concurrent with an increase in illicit substance purchasing opportunities, frequent use of cannabis may also lead to assimilation into deviant and substance using peer-groups which in turn may lead to an escalation in illegal drug use [14, 41]. As some jurisdictions have commenced legalisation of cannabis, it will be interesting to observe whether the strong correlation between patterns of cannabis use and the use of stimulants persist in those settings. There is also evidence that neuropsychological changes may link increased cannabis use to the use of stimulants; neuroimaging studies suggest shared neurological pathways that may increase the risk for later and wider substance misuse [11, 42–44].

Findings from our study identified a subgroup of cannabis users–individuals with a CUD–that are vulnerable not only to the risks associated with cannabis abuse or dependence, but also with the additional risks associated with the use of stimulants. Importantly, cannabis use in the absence of other illicit drugs has been found to have fewer adverse effects on social relationships, physical health, work, educational studies/employment opportunities and financial position than when cannabis is used with other substances [45]. It is possible that these problematic cannabis users represent a subset of individuals who are using cannabis to self-medicate in order to mitigate distressing symptoms, including anxiety and depression, possibly precipitated by the use of stimulants. As problematic use of drugs may interfere with the function of neurotransmitters and compromise decision-making, polysubstance use to augment perceived self-medication may place individuals with a CUD at further risk of negative health consequences [11].

Cannabis and stimulants can each cause adverse cardiovascular symptoms while the combination of these different drug classes may increase the likelihood of such outcomes [16, 17]. In addition, both drug classes can precipitate adverse mental health symptoms and cannabis and stimulants are frequent contributors to drug-related psychiatric admissions in Ireland [46]. Again, the combination of both classes within the one person is likely to complicate such presentations [47]. Given the increased risk of both physical and psychological harm associated with polydrug use, it will be important to monitor these relationships in Ireland and to develop harm reduction initiatives in order to reduce polydrug use and avert harm [5]. The frequent co-use of these drug classes also has important implications in addiction treatment, where it will be important to explore use of the alternate class of drugs in instances where clients present with a use disorder related to the other class.

### Strengths and limitations

This research has several strengths. This is the first study to examine relationships between patterns of cannabis use and recent use of stimulants in Ireland using data from two population surveys. A further strength of this research is the large sample size and that respondents were selected using random probability sampling that was representative of the Irish population; thus, our findings are generalisable to the whole population. We also controlled for important potential confounders in analyses and used valid and reliable measures of cannabis abuse and dependence, defined using the DSM-IV. Research on relationships between patterns of cannabis use and the use of stimulants is important for preventative work and for informing and assessing drug policy. This is particularly relevant at the present time given the debate regarding the decriminalisation of cannabis in many countries.

Despite these strengths, several limitations should be noted. The cross-sectional study design limits inference with regard to causality and precludes drawing conclusions regarding the temporal direction of relationships. Consequently, while it is highly plausible that cannabis is a gateway drug, causal links cannot be examined in the current datasets. Nevertheless, while a cross-sectional study has reduced ability to identify direction of causality in relationships, it has the advantage of including a much wider age range than typical prospective studies. In addition, this work highlights the scale and extent of polydrug use in Ireland and is highly relevant for both clinicians and other practitioners caring for substance misusers and for policy makers. Another limitation of this research is the use of self-reported questionnaires which are subject to potential inaccuracies, recall and reporting bias. Therefore, residual confounding arising from imprecise measurement of variables should also be considered. In addition, although we speculate that covariates of mental health may be a significant driver between CUD and recent use of stimulants, we did not have data on psychiatric disorders for either survey to allow us to explore this. There is also a possibility that relationships between cannabis use and stimulants such as methamphetamines or other prescribed stimulant drugs may also exist. Nevertheless, our combined surveys showed low-level use of these drugs and we chose to concentrate our analyses on cocaine and ecstasy given the notable increase in use of these substances in Ireland over recent years. Also, due to sample size constraints, we analysed cannabis abuse and dependence together rather than as separate constructs. Research examining abuse and dependence separately may be warranted as abuse or dependence criteria may be differentially predictive of adverse outcomes.

## Conclusions

In conclusion, the results from this study suggest a relationship between patterns and frequency of cannabis use and recent use of stimulants and an association between CUD and recent use of cocaine or ecstasy. Health professionals should be aware of these relationships. As the use of cannabis with stimulants may increase the risk of negative health consequences, education in community and medical settings about polydrug use and its increased risks may be warranted. Findings from this study may be used to better inform public health efforts to improve prevention of problematic drug use in the Irish and other populations and in the identification and referral of clients to appropriate treatment services.

## Supporting information

S1 Dataset The 2010/11 and 2014/15 Irish National Drug Prevalence Surveys dataset. (SAV)
